# Synthesis, Functionalization, and Biomedical Applications of Iron Oxide Nanoparticles (IONPs)

**DOI:** 10.3390/jfb15110340

**Published:** 2024-11-12

**Authors:** Mostafa Salehirozveh, Parisa Dehghani, Ivan Mijakovic

**Affiliations:** 1 Systems and Synthetic Biology Division, Department of Life Sciences, Chalmers University of Technology, SE-412 96 Gothenburg, Sweden; mostafas@chalmers.se; 2 James Watt School of Engineering, University of Glasgow, Glasgow G12 8QQ, UK; parisa.dehghani@glasgow.ac.uk; 3 The Novo Nordisk Foundation Center for Biosustainability, Technical University of Denmark, DK-2800 Kongens Lyngby, Denmark

**Keywords:** iron oxide, synthesis, functionalization, biomedical, biosensor

## Abstract

Iron oxide nanoparticles (IONPs) have garnered significant attention in biomedical applications due to their unique magnetic properties, biocompatibility, and versatility. This review comprehensively examines the synthesis methods, surface functionalization techniques, and diverse biomedical applications of IONPs. Various chemical and physical synthesis techniques, including coprecipitation, sol–gel processes, thermal decomposition, hydrothermal synthesis, and sonochemical routes, are discussed in detail, highlighting their advantages and limitations. Surface functionalization strategies, such as ligand exchange, encapsulation, and silanization, are explored to enhance the biocompatibility and functionality of IONPs. Special emphasis is placed on the role of IONPs in biosensing technologies, where their magnetic and optical properties enable significant advancements, including in surface-enhanced Raman scattering (SERS)-based biosensors, fluorescence biosensors, and field-effect transistor (FET) biosensors. The review explores how IONPs enhance sensitivity and selectivity in detecting biomolecules, demonstrating their potential for point-of-care diagnostics. Additionally, biomedical applications such as magnetic resonance imaging (MRI), targeted drug delivery, tissue engineering, and stem cell tracking are discussed. The challenges and future perspectives in the clinical translation of IONPs are also addressed, emphasizing the need for further research to optimize their properties and ensure safety and efficacy in medical applications. This review aims to provide a comprehensive understanding of the current state and future potential of IONPs in both biosensing and broader biomedical fields.

## 1. Introduction

**Figure 1:**
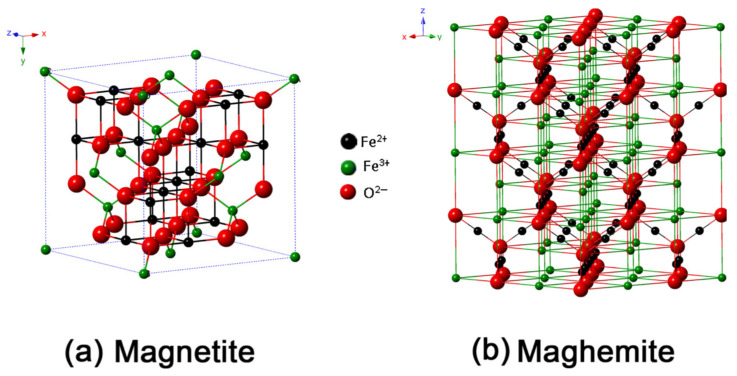
The crystal structure of (**a**) magnetite and (**b**) maghemite, where Fe^2+^ ions are represented by black spheres, Fe^3+^ ions by green spheres, and O^2−^ ions by red spheres. Reprinted from reference [2].

Iron is an essential trace element in the human body, playing a critical role in numerous biological functions [1]. Iron oxides are particularly significant among the various iron-based compounds [2]. These oxides are typically classified into three categories: oxides, hydroxides, and oxyhydroxides [3]. Due to the unique properties and specific physicochemical characteristics of iron oxide nanoparticles (IONPs), they are utilized in biomedical fields in the forms of γ-Fe_2_O_3_, α-Fe_2_O_3_ and Fe_3_O_4_ (Figure 1) [4]. Figure 1a shows that Fe_3_O_4_ has a face-centered cubic inverse spinel structure with a cubic close-packed array of O^2−^ ions along the (111) orientation, the plane of the face of the crystal. In this structure, Fe^2+^ ions occupy half the octahedral sites, while Fe^3+^ ions split between the remaining octahedral and tetrahedral sites, giving Fe_3_O_4_ the lowest resistivity among iron oxides due to its small bandgap (0.1 eV) [5]. Figure 1b depicts γ-Fe_2_O_3_, which also has a cubic structure with 32 O^2−^ ions, 21⅓ Fe^3+^ ions, and 2⅓ vacancies. The maghemite structure, a fully oxidized form of magnetite, is an n-type semiconductor with a 2.0 eV bandgap [2]. The quantum size phenomena can impart unique optical and electrical characteristics, as well as high magnetic behavior, to the IONPs [6,7]. Consequently, magnetite (Fe_3_O_4_) and maghemite (γ-Fe_2_O_3_) exhibit enhanced superparamagnetic properties within the size range of 10–20 nm [8]. These nanoparticles are highly valued for their unique properties, including biodegradability, biocompatibility, cost-effectiveness, and environmental safety.

These attributes render them highly suitable for a range of biomedical applications, including drug delivery, tissue engineering, biosensing, MRI (magnetic resonance imaging), stem cell therapy, and cancer treatment [9,10,11]. In contrast, iron oxide composites incorporating other magnetic elements (e.g., MnFe_2_O_4_ and CoFe_2_O_4_) or metal alloys (such as FeCo and FePt) exhibit superior magnetic properties. However, due to their inherent toxicity and susceptibility to rapid oxidation, their use in biological systems is significantly limited [12]. Despite the promising potential of IONPs, bare iron oxide nanoparticles have been shown to exhibit cytotoxic effects in vitro.

**Figure 2:**
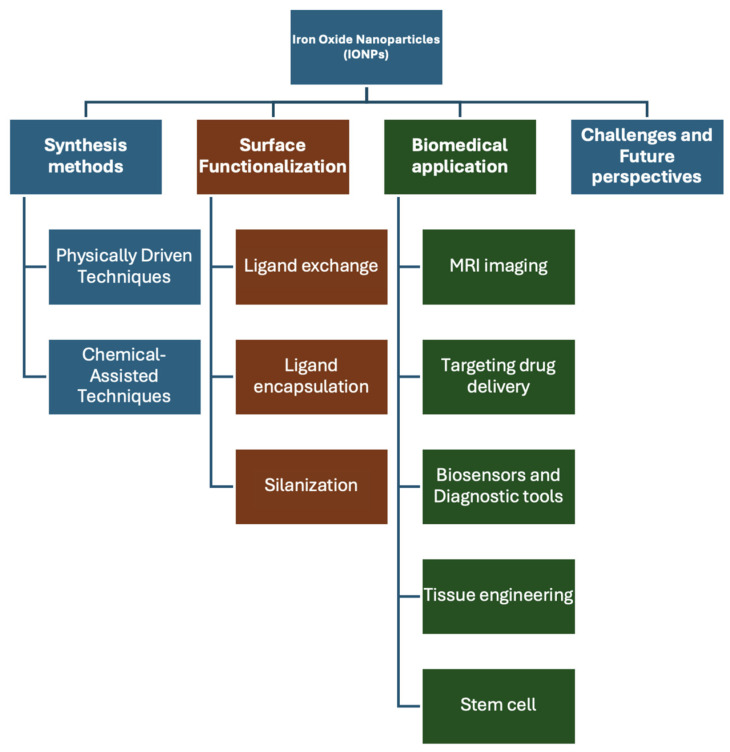
An overview diagram of this paper including the synthesis, functionalization, and biomedical applications of iron oxide nanoparticles (IONPs).

Therefore, surface modification or encapsulation with biocompatible materials is essential to mitigate cytotoxicity and enhance their compatibility with human cells. The design of IONPs for biomedical applications requires precise control over particle size and surface coating, as these parameters critically influence their behavior and efficacy in biological environments [13,14,15,16]. This review paper provides an overview of iron oxide nanoparticle synthesis, functionalization, and biomedical applications. It covers various synthesis methods, including chemical-assisted techniques like coprecipitation and sol–gel processes, as well as green synthesis and physically driven methods such as aerosol techniques and laser ablation. The review also addresses surface functionalization strategies and explores applications in MRI, targeted drug delivery, tissue engineering, stem cell research, and biosensing. Additionally, it discusses challenges and future perspectives in the field. An overview diagram summarizing these concepts is presented in Figure 2.

## 2. Synthesis Methods

**Figure 3:**
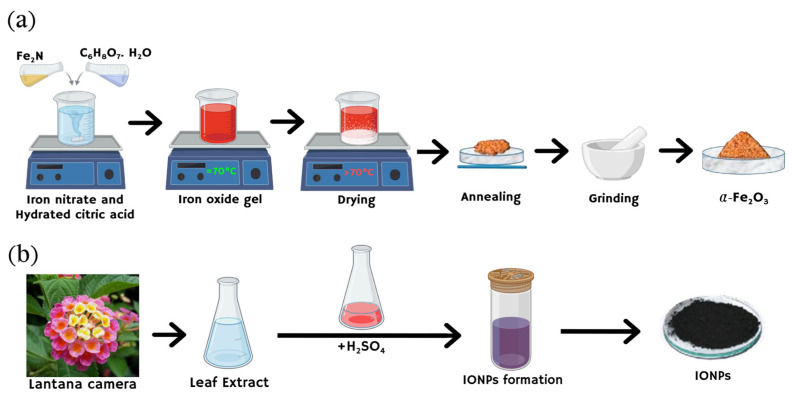
A schematic illustration of the synthesis of IONPs. (**a**) The synthesis of iron oxide nanoparticles via the sol–gel method: iron nitrate and citric acid are mixed, forming an iron oxide gel, followed by drying, annealing, and grinding to obtain α-Fe_2_O_3_ nanoparticles. (**b**) The synthesis of iron oxide nanoparticles via the green chemistry method: ferrous sulfate is combined with plant extract and sodium hydroxide, centrifuged, and oven-dried to produce a brownish-black powder for storage [29,30].

In the last twenty years, numerous physical, chemical, and biological techniques have been investigated to synthesize IONPs with precise control over their size and morphology [17,18,19,20,21]. The performance of IONPs is significantly determined by their nanoscale dimensions, usually with adjustable sizes under 50 nm, a spherical structure, a high surface area, and superparamagnetic qualities [22,23]. Each synthesis approach provides unique control over properties such as particle shape, size, distribution, chemical stability, and magnetic characteristics. Achieving precise control over the dimensions and morphology of iron oxide nanoparticles (IONPs) remains a formidable challenge. Nevertheless, wet chemical methodologies are prevalently utilized due to their capacity to produce nanoparticles with exceptional purity [24,25,26,27]. Notably, the coprecipitation technique is extensively favored for its straightforward procedural approach and its efficacy in generating IONPs with optimal characteristics for biomedical applications [28]. In the following section, these methods are reviewed, with Figure 3 highlighting the most common approaches to synthesizing IONPs using both the sol–gel and green chemistry methods.

### 2.1. Chemical-Assisted Techniques

#### 2.1.1. Coprecipitation

**Figure 4:**
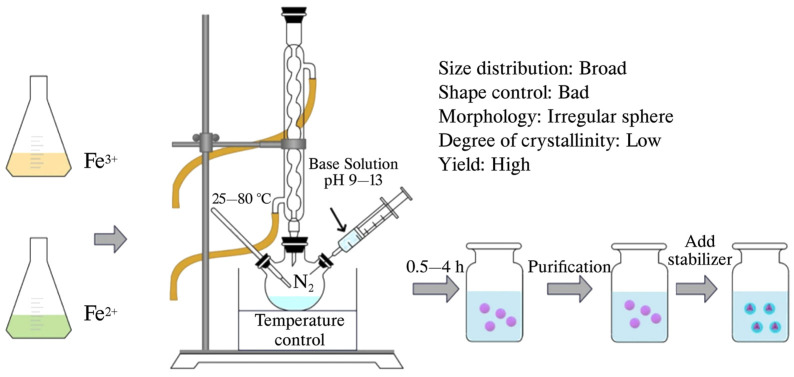
Schematic illustration of IONPs synthesis via coprecipitation technique. Reprinted from reference [49].

The coprecipitation method is highly regarded as a leading technique for synthesizing IONPs, especially in biomedical applications, owing to the generally non-toxic nature of the materials used [31]. This approach involves the simultaneous precipitation of Fe(II) and Fe(III) salts in an alkaline aqueous environment, producing high-purity magnetic nanoparticles with desirable size and morphology at low temperatures (Figure 4) [32]. Specifically, magnetite (Fe_3_O_4_) is favored for its stability, strong ferromagnetic properties, and non-toxicity [20]. The process is conducted in an inert nitrogen atmosphere at room temperature, allowing for the production of spherical IONPs with diameters ranging from 5 to 40 nm [28,33,34]. Oxygen is vital in the formation of magnetite, making the use of nitrogen gas to eliminate oxygen highly effective. To control the size and shape of the nanoparticles, it is crucial to carefully manage various reaction parameters [35]. The coprecipitation method is the most straightforward and effective approach for synthesizing Fe_3_O_4_, with a high success rate ranging from 96 to 99.9% [36]. The chemical reaction for magnetite formation is represented as follows:

Fe^2+^ + 2 Fe^3+^ + 8 OH^−^ → Fe_3_O_4_ + 4H_2_O

This technique relies on the precise control of parameters such as metallic precursor types, pH, ionic strength, and reaction temperature to regulate the nanoparticles’ size, shape, and magnetic properties [37,38]. The nucleation of magnetite can be regulated by maintaining the pH within a specific range, with higher pH levels promoting the growth of the nuclei [39]. The coprecipitation method is particularly advantageous for biomedical applications due to its ability to produce IONPs at a large scale and its direct yield of water-soluble IONPs [40,41]. For reducing nanoparticle size below 3 nm, citrate ions are often used, although this can lead to a decrease in the magnetic properties of the IONPs due to reduced crystallinity [42]. To improve size and shape, researchers have modified the coprecipitation method by coating the IONPs with polymers, which reduces the size to approximately 7.2 nm and enhances the spherical shape compared to uncoated particles, also improving their superparamagnetic properties [43,44]. Additionally, altering the medium to include alkaline amines can enhance magnetic properties and reduce particle size, addressing the challenge of maintaining magnetic performance with smaller sizes [45,46,47]. The coprecipitation method is especially valued for its simplicity and cost-effectiveness, which facilitate large-scale production and the manufacture of commercial MRI contrast agents like Ferucarbotran, Feridex, and Combidex [10]. However, challenges persist, including difficulties in controlling nanoparticle size distribution and achieving high crystallinity and optimal magnetic characteristics, affected by kinetic factors and the low synthesis temperature [48].

#### 2.1.2. Sol–Gel Process

**Figure 5:**
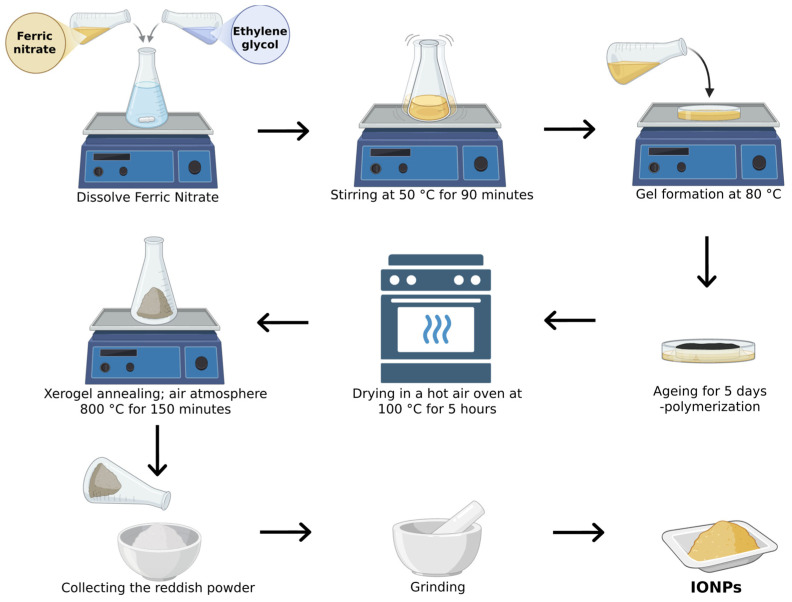
Schematic illustration of IONPs synthesis via sol–gel technique.

This technique for producing IONPs involves the hydroxylation and condensation of certain iron-based precursors, creating a “sol” (or a colloidal solution) of nanoparticles, which is subsequently dried or ‘‘gelled’’ by removing the solvent until a 3D iron oxide network is formed [50,51]. As illustrated in Figure 5, a 0.15 M solution of ferric nitrate nonahydrate was prepared by dissolving it in 50 mL of ethylene glycol at 50 °C, stirred for 90 min to ensure homogeneity. The temperature was then raised to 80 °C with continuous stirring until a brown semi-solid gel formed, which was aged for 5 days at room temperature. After aging, the gel was dried at 100 °C for 5 h to obtain a solid xerogel, which was then annealed at 800 °C for 150 min to yield iron oxide nanoparticles. The final product was cooled and ground into a fine reddish powder [52]. The size of the resulting IONPs can be adjusted between 15 and 50 nm [53]. The sol–gel approach is a crucial method for synthesizing IONPs [54]. In this process, starting from molecular precursors such as metallic alkoxides or inorganic salts, an oxide framework is produced through hydrolysis and polymerization reactions at relatively low temperatures, allowing for the creation of metastable oxide phases [55,56]. The sol–gel method offers numerous benefits, such as the use of less expensive precursors compared to alkoxides, which are quite costly, and a straightforward preparation process that yields nanoscale particles with uniform size distribution [57]. Additionally, this method enables the production of high-purity, consistent nanomaterials at lower temperatures. However, as highlighted by other researchers, the primary drawbacks include the use of organic solvents and the fact that the procedure involves multiple stages, making it time-intensive and expensive [58]. Consequently, some studies have modified the sol–gel method to minimize reaction time, reduce the number of procedural steps, and avoid using organic solvents where, for instance, the direct use of water-soluble polymers can remove the need for the polymerization step [59]. This method still generates pollution from reaction by-products and requires further processing of the final products. Based on our observations, even with these modifications, the sol–gel method cannot be considered a replacement for the green synthesis approach, as the use of harmful chemicals remains unavoidable. 

#### 2.1.3. Thermal Breakdown

**Figure 6:**
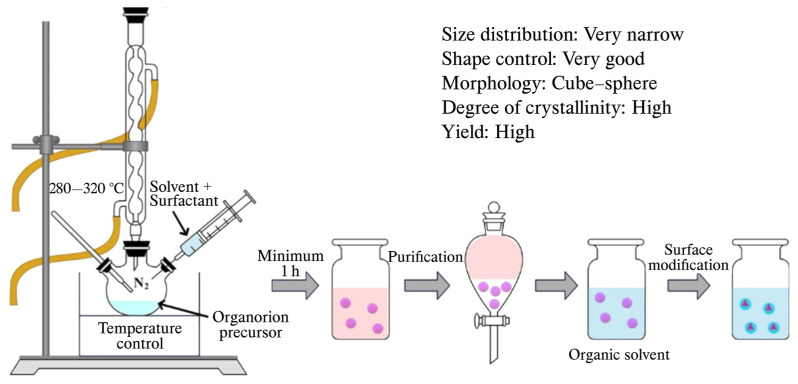
Schematic illustration of IONPs synthesis via thermal breakdown technique. Reprinted from reference [49].

The synthesis of small, uniform IONPs is crucial for their application in medicine, as size and uniformity significantly influence their properties. Traditional methods like coprecipitation often face issues with scaling and low crystallinity [60]. In the heating-up method (Figure 6), a mix of organometallic precursors, surfactants, and solvents is gradually heated until nanoparticles form and grow. In contrast, the hot-injection technique quickly adds chemicals to a heated surfactant solution to trigger nucleation, then controls growth [61]. Key variables like reagent ratios, temperature, and duration of the reaction play a significant role in determining the properties of the nanoparticles [62,63]. Typically, iron carbonyl or acetylacetonates serve as precursors, while stabilizing agents such as fatty acids or oleic acid prevent the nanoparticles from aggregating [64]. Maintaining an inert environment, often achieved using argon, is essential during the reaction, which occurs within a temperature range of 100–350 °C, to ensure that the particles are highly crystalline and consistent in size [65].

Krishnan et al. synthesized monodisperse IONPs using three iron precursors and demonstrated that increasing the concentration of surfactants leads to larger, yet highly uniform particles [62]. Despite its advantages in producing high-quality IONPs, the thermal decomposition method is limited for large-scale production due to the toxicity of the reagents, high costs, and process complexity. However, Bergstrom et al. achieved size control between 5 and 27 nm by employing oleic acid and sodium oleate to prepare iron oleate precursors, fine-tuning the size and shape through adjustments in reflux time, temperature, and surfactant concentration [66]. To overcome the scalability issue, Hyeon et al. developed a cost-effective and non-toxic approach using iron chloride, synthesizing 40 g of IONPs with size variation below 5% without any size-sorting step, making it viable for large-scale production [67]. Additionally, Fortner et al. demonstrated the synthesis of highly crystalline nanocrystals with sizes ranging from 8 to 40 nm by carefully adjusting reactant ratios, time, and co-surfactant concentrations, achieving a high monodispersity of less than 10% [68]. This variation in the thermal decomposition method underscores its flexibility and adaptability in producing IONPs with controlled size and high crystallinity, though further developments are needed to fully optimize the process for scalable production.

#### 2.1.4. Hydrothermal Synthesis

The hydrothermal method, along with its various adaptations, is widely used to produce crystalline IONPs that exhibit consistent size and shape. Typically, this process involves dissolving solid metal linoleate in an aqueous ethanol-linoleic acid solution, which is then subjected to high temperatures (around 220 °C) and pressures greater than 107 Pa for about 72 h inside a Teflon-lined autoclave [64]. This approach enables precise control over the particle size, ranging from just a few nanometers to several hundred, with smaller particles, typically under 80 nm, being preferred for their enhanced magnetic properties [69]. Precursor concentration, reaction duration, and temperature play essential roles in influencing the size and crystallinity of the particles. Furthermore, hydrothermal synthesis is environmentally friendly, as it eliminates the need for organic solvents or post-synthesis treatments, while simultaneously enabling surface modification through functional ligands to improve hydrophilicity and dispersibility for biomedical purposes [70,71]. To enhance production efficiency and minimize inconsistencies between batches, continuous flow hydrothermal synthesis has been implemented. This method facilitates the production of uniform IONPs with a range of sizes (4–100 nm) and diverse shapes [72,73].

The hydrothermal microwave technique employed for the synthesis of IONPs amalgamates the benefits inherent in hydrothermal synthesis with microwave irradiation to bolster the efficiency and regulation of nanoparticle fabrication [74,75]. This approach exploits microwave energy, thereby facilitating swift and uniform thermal distribution within the reaction mixture, culminating in reduced reaction durations and enhanced product yields relative to traditional synthesis methodologies [76]. Within hydrothermal frameworks, iron oxide precursors are exposed to elevated temperatures and pressures within a hermetically sealed environment, which promotes the crystallization and development of nanoparticles. The incorporation of microwave energy facilitates in-core volumetric heating, which assists in attaining a more uniform temperature distribution, thereby minimizing side reactions and augmenting the characteristics of the resultant nanoparticles [77]. In summary, the hydrothermal microwave methodology signifies a promising and effective strategy for the synthesis of IONPs, providing the opportunity for expedited experimentation while ensuring enhanced regulation over the synthesis parameters and characteristics of the final product. The application of a microwave-assisted hydrothermal method for the fabrication of IONPs exemplifies an innovative methodology that significantly augments both the efficiency and efficacy of the production process. This technique leverages the rapid and homogeneous thermal characteristics of microwave radiation, thereby enabling meticulous regulation of reaction parameters and resulting in the generation of iron oxide-hydrochar (FHC) and iron oxide-activated hydrochar (FAC) composites. The distinctive architecture of these composite materials, which incorporates crystalline iron oxide within an amorphous hydrochar matrix, markedly enhances the adsorption capacity for contaminants, particularly in the elimination of methylene blue dye from aqueous solutions [78]. This novel synthesis methodology not only mitigates the challenges related to the recovery of adsorbents following treatment but also presents a viable solution for the development of sustainable and efficient adsorbent materials for environmental remediation. By leveraging the magnetic characteristics of IONPs alongside the porous configuration of hydrochar, this strategy facilitates progress in adsorption technologies, thereby offering a novel approach to combat aquatic pollution and illustrating the potential of microwave-assisted hydrothermal method in the fabrication of multifunctional nanomaterials [79].

#### 2.1.5. Sonochemical Route

A novel approach for synthesizing IONPs is the sonochemical method [80]. In this process, intense ultrasound waves generate acoustic cavitation, creating hot spots with temperatures as high as 5000 K and pressures close to 1800 atm [81]. The extreme heat from these localized hot spots causes bubbles to collapse inward, which leads to the formation of IONPs. This technique also allows for the creation of uniform nanoparticles with a variety of shapes [82]. Additionally, the sonochemical approach is suitable for producing large quantities of nanoparticles [83]. In 2014, Ghanbari and colleagues successfully synthesized Fe_3_O_4_ nanoparticles through a simple sonochemical reaction conducted without the use of surfactants, at room temperature, and in the absence of an inert gas [84]. Their study also examined the impact of different parameters on the shape and structure of the nanoparticles. In this procedure, the mixture was subjected to ultrasonic waves for 1 h, with continuous mechanical stirring. A Bandelin Multiwave Ultrasonic Generator (model MS 72) from Berlin, Germany, equipped with a converter/transducer and titanium oscillator, was used to deliver ultrasonic irradiation at a frequency of 20 kHz and a maximum power output of 76 W. After sonication, the material was dried at 105 °C for 24 h. The resulting product underwent an 8-step tapping process, yielding approximately 15% magnetite. While this method can enhance the crystallinity and magnetic properties of Fe_3_O_4_ nanoparticles, large-scale production requires substantial energy input, along with high temperatures and pressures, to maintain uniformity in particle size.

#### 2.1.6. Microemulsion Method

**Figure 7:**
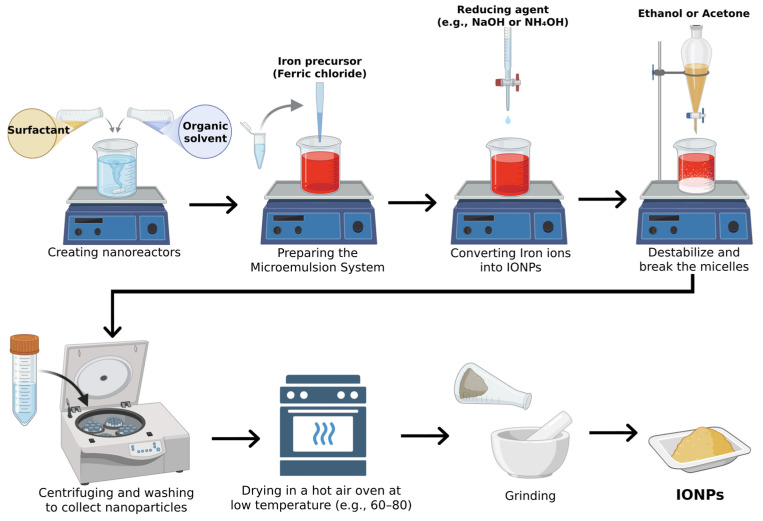
Schematic illustration of IONPs synthesis via microemulsion technique.

The microemulsion method, commonly known as the water-in-oil (W/O) or reverse micelle technique, employs water droplets in an organic phase as nanoreactors, stabilized by surfactant molecules, to achieve controlled crystal growth of IONPs [85]. The iron precursor solution is dispersed in a water-in-oil (W/O) microemulsion, where water nanodroplets are stabilized by surfactants in an organic solvent. A reducing agent is then added dropwise, initiating nanoparticle formation within these droplets. After stirring for 1–2 h, the microemulsion is destabilized with ethanol or acetone, and centrifugation is used to collect the iron oxide nanoparticles. The particles are thoroughly washed to remove residual chemicals and then dried for storage (Figure 7) [86]. In this process, iron precursors are precipitated in the aqueous phase inside the micelles, while the organic phase prevents unwanted precipitation since the precursors are inactive at this stage [87]. By adjusting the size of water droplets and selecting appropriate precursors, the size and morphology of the resulting nanoparticles can be fine-tuned (Figure 7) [88,89]. Studies have shown that maghemite nanoparticles (γ-Fe_2_O_3_) with sizes ranging from 10 to 25 nm and a spherical, nearly monodispersed shape can be synthesized using this method [90,91]. Nevertheless, notwithstanding its benefits in regulating nanoparticle synthesis through the employment of surfactants as nanoreactors to modulate both nucleation and crystal growth, the microemulsion technique is beset by considerable limitations, which encompass being labor-intensive, costly, and not eco-friendly [92,93,94]. Additionally, for applications like water treatment, it is essential to ensure that the nanoparticles synthesized can disperse in an aqueous medium with minimal toxicity.

#### 2.1.7. Polyol Method

The polyol method is a versatile and scalable approach for producing uniform IONPs in hydrophilic polyalcoholic solvents like ethylene glycol, triethylene glycol, and polyethylene glycol (PEG) [95]. These solvents act as high-boiling point solvents, reducing agents, and stabilizers, preventing nanoparticle aggregation and controlling their growth. The method is conducted at high temperatures, ensuring high crystallinity and strong magnetic properties in the resulting nanoparticles, which also feature a hydrophilic coating, simplifying their use in biological applications [96]. The polyol method involves the reduction in metal salts (such as nitrite, oxide, chloride, acetate, and acetylacetonate) into IONPs using polyol, which acts as both a surfactant and a reducing agent. This technique allows for controlled particle growth while preventing agglomeration. The type of polyol, concentration of metal salts, and temperature are critical factors that influence the size and shape of the resulting particles [92]. However, the reaction conditions have so far been largely empirical, limiting reproducibility and scalability. For example, a wide range of nanoparticle sizes, from 4 to 100 nm, are produced depending on parameters such as temperature, reaction time, and solvent choice [97,98]. Although the polyol method offers advantages like cost-effectiveness without the need for an inert atmosphere, it faces challenges such as the requirement for high temperatures and pressures, and difficulties in producing particles smaller than 10 nm [99,100,101]. This highlights the need for further studies of the nucleation and growth mechanisms. The microemulsion method uses surfactants as nanoreactors to control nucleation and crystal growth, while the sol–gel method involves the hydrolysis of metal alkoxides to produce IONPs with good shape control but low crystallinity.

### 2.2. Physically Driven Techniques

#### 2.2.1. Aerosol/Vapor-Phase Methods

Gas-phase deposition techniques, like Chemical Vapor Deposition (CVD) and Physical Vapor Deposition (PVD), are highly efficient for creating IONPs [102]. In PVD, precursor molecules in gas form are supersaturated or condensed thermally onto a surface, generating fine, though sometimes inconsistent, IONPs. In contrast, CVD is known for producing high-quality thin films or nanotubes with increased purity, as contaminants are less of a concern. Despite its suitability for mass production, maintaining uniform nanoparticle size can be challenging [103]. Additionally, decomposition reactions involving precursors like iron trifluoroacetylacetonate or acetylacetone at temperatures of 300 °C or 400–500 °C yield IONPs, although further reduction steps may be needed. This approach may also lead to variability in particle size, crystallinity, and other properties [104].

On the other hand, the aerosol/vapor method divides into flame spray, and laser pyrolysis techniques depend on flame reactors which provide IONPs in high yield [105,106] with high rate in synthesis of IONPs [107,108]. This method synthesizes three kinds of IONPs such as maghemite (Fe_2_O_3_), magnetite (Fe_3_O_4_), and wustite (FeO) which depend on different ratios of fuel to air within combustion and valance states of iron precursor when applied [106]. Spray pyrolysis involves the use of ferric salts and reductant in a carbon-based solvent that is sprayed into reactors during the pyrolysis process in order to amass the aerosol solute and evaporate the solvent [107,109,110]. The size of the resulting particles depends on the initial size of the droplets [111]. By using various iron salts in water and ethanol, it is possible to control the size and shape of Fe(II)-deficient magnetite IONPs [112]. A single-step laser pyrolysis method can reduce the volume of the reaction. In this technique, laser is used in order to heat the gaseous mixture of an iron precursor and a flowing mixture of gasses to obtain homogenous particles which have significant characteristics such as small and narrow size and are well-dispersed without aggregation of nanoparticles [107]. In this technique, no solvent is used. By adjusting the condition of the reaction, the NPs’ sizes can be controlled between 2 and 7 nm [113]. The spray drying process of a hybrid silica/spinel Fe_3_O_4_ prepared a multifunctional platform for biomedical application such as drug delivery, MRI contrast agent, and hyperthermia which allowed us to choose the size and quantity of IONPs [114]. Physical deposition techniques such as thermal evaporation and sputtering are simple but costly and produce low volumes of material, necessitating improvements for industrial scale-up.

#### 2.2.2. Pulse Laser Ablation

This approach to nanoparticle synthesis is notable for its ease of use and presents a promising option for generating nanoparticles with precisely controlled variables like pressure and temperature that are harder to manage in other methods. During the ablation phase, high temperatures and pressures produce a plasma plume containing ionized material from both the solvent and the target. These particles then interact with the ablated material, undergoing nucleation and growth processes to form metastable nanoparticles [115]. The simplicity, speed, and cost-effectiveness of this method have made it more successful than other nanoparticle synthesis techniques [116]. Furthermore, it avoids harmful chemical exposure and does not produce toxic by-products, unlike many traditional chemical processes [117,118]. The laser ablation technique operates by removing material from an iron source using a laser beam with specified parameters like intensity, wavelength, and diameter, which is directed onto the bulk substrate within a chosen solvent medium to synthesize IONPs. Several studies have demonstrated this process, using six different HPLC-grade solvents such as acetonitrile, tetrahydrofuran, dimethylformamide, dimethyl sulfoxide, toluene, and ethanol combined with iron precursors to produce nanoparticles around 15 nm in size. Water has also been used with similar results [119,120,121] to SDS [122], ethanol [123], acetone [120], poly-(vinylpyrrolidone) [115], oleic acid, and oleylamine [124]. Although laser ablation has gained popularity, it does face challenges due to technical issues and complications related to the ablation process itself. The high kinetic energy of certain species may lead to re-sputtering, and an uneven energy distribution in the laser beam may cause an inconsistent energy profile in the plasma plume [125].

#### 2.2.3. Biomimetic/Green Synthesis Techniques

The green or biological synthesis of IONPs presents an eco-friendly alternative to conventional methods, avoiding toxic materials and hazardous by-products. This approach uses natural materials, such as plant extracts, bacteria, fungi, algae, and yeasts, which serve as reducing and capping agents to stabilize and control the size and morphology of the nanoparticles [126]. Biological methods can be divided into biologically induced biomineralization, where nanoparticles form naturally in the culture solution, and biologically controlled biomineralization, where synthesis occurs intracellularly under controlled conditions [127]. For example, plant extracts like those from *Rhus coriaria* and *Moringa oleifera* have been used to produce nanoparticles with desirable properties, such as antibacterial activity [128]. Similarly, microorganisms and proteins, such as ferritin, have been utilized to create well-ordered and uniform nanoparticles. Biomineralization involves using living organisms to form and store inorganic minerals, yielding particles from nanometers to centimeters in size [129,130]. This process can be biologically induced and controlled, with proteins serving as templates to control particle size. For instance, magnetosomes, which are iron-rich particles produced by bacteria and algae, typically range from 50 to 100 nm and have various applications due to their high iron concentration [131,132]. Magnetosome has a high concentration of iron which is a good reason to use it in many applications [133]. Accordingly, Hu et al. synthesized IONPs by using pluronic F127 as a mineralization template and peptide-WSG as tumor-specific bioligand. The IONPs’ shape was sphere-like and uniform with sizes between 30 and 40 nm [134]. Despite the benefits of green synthesis, including simplicity, cost-effectiveness, and reduced waste, challenges such as particle stability, uniformity, and scalability remain.

## 3. Surface Functionalization

**Figure 8:**
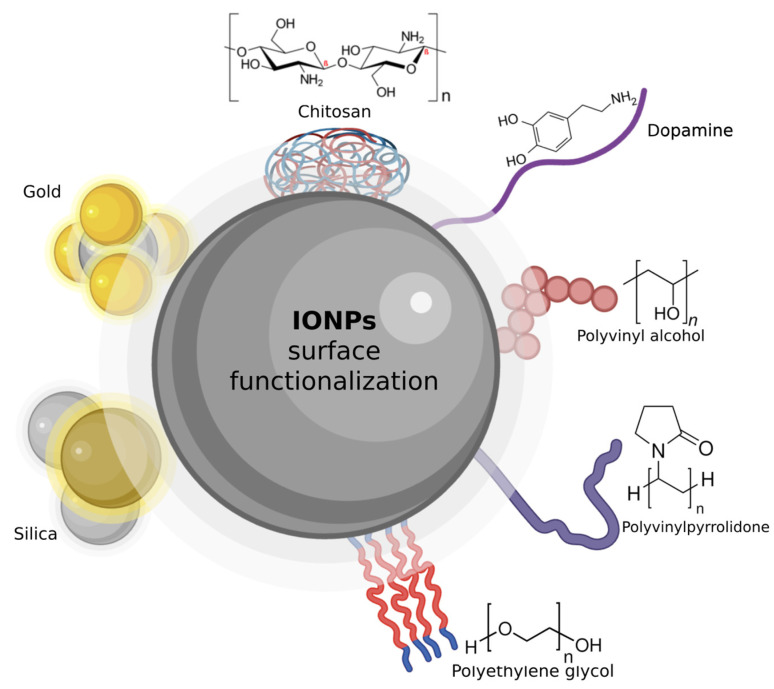
Multiple surface functionalizations of magnetic IONPs.

IONPs are often produced with hydrophobic coatings, making them unsuitable for direct use in aqueous biological environments. To enhance their biocompatibility and functionality, surface modification is essential [135]. This process involves coating the nanoparticles with water-solubilizers, such as inorganic or organic small molecules or macromolecules, which can improve their dispersion and stability in water [136]. Surface engineering also allows for the introduction of tailored functions, like tissue targeting, environmental responsiveness, and therapeutic delivery. This modification can be performed either during synthesis or afterwards, utilizing chemical function motifs that bind strongly to hydroxides on the nanoparticle surface (Figure 8).

On the other hand, innovative strategies for functionalization unveil their underlying principles, benefits, and results in MRI, drug delivery, and catalysis, which stand out as key areas for surface-modified IONPs. Table 1 summarizes the most prevalent techniques employed to functionalize IONPs, categorized by their coating type (organic or inorganic), specific precursor materials, and their advantages and disadvantages. The modification of the IONPs’ surfaces is a crucial phase that bestows upon them unique functionalities, thus paving the way for their application across various fields. Numerous strategies have been employed, each showcasing unique merits and drawbacks. For biomedical applications, IONPs are favored due to their biocompatibility, unique properties, and chemical stability. However, to ensure they remain stable in biological environments, modifications are necessary. Coating with biocompatible shells can prevent issues such as oxidation, aggregation, and non-specific interactions with serum proteins, which are common with naked IONPs. Various polymers and monomers are used for surface modification, with the choice of the coating layer influencing the nanoparticles’ dispersion, stability, and overall performance [137]. Advanced polymers are particularly beneficial due to their hydrophilicity, biocompatibility, and versatility, significantly impacting the properties and applications of IONPs in biomedical fields (Table 2).

### 3.1. Ligand Exchange

Ligand exchange is a prevalent method for modifying the surface properties of IONPs, converting their hydrophobic nature to hydrophilic, and causing chemical bonding between IONPs and functional groups. This process involves replacing the initial hydrocarbon layer with new functional groups that both bonds tightly to the IONP surface and enhances water solubility. By using ligands such as amines, carboxylic acids, dopamine, or phosphine the colloidal stability of the nanoparticles is achieved [148,149]. For instance, Dong et al. utilized nitrosyl tetrafluoroborate to exchange organic ligands, preserving the nanoparticles’ size and shape while maintaining stability in hydrophilic media [150]. Similarly, Wan et al. applied sodium tripolyphosphate (STTP) to IONPs for improved stability in phosphate-buffered saline (PBS) and used them for MRI due to their low toxicity and optimal T1 relaxation [151]. Ligand exchange offers versatility and can be performed under mild conditions, but it may face challenges such as limited long-term stability and difficulties in controlling shell thickness.

### 3.2. Ligand Encapsulation

For biomedical application, one of the other approaches to creating hydrophilic and biocompatible IONPs is ligand encapsulation which encapsulates nanoparticle in self-assembled polymer. In this innovative approach, IONP creation unfolds through a series of connections with an encapsulating agent that resides within the solution. 

The art of in situ encapsulation utilizing polymeric materials and metals, including polyethylene glycol and gold, has been vividly illustrated. Additionally, Fe_3_O_4_ nanoparticles were encapsulated inside the organic alginate with D-galactosamine as a cell-targeting ligands and produced a core–shell structure (Fe_3_O_4_@Alg-GA) [152]. A diverse collection of IONPs were synthesized via in situ coprecipitation of ferrous (Fe^2+^) and ferric (Fe^3+^) from aqueous solutions [153]. Additionally, various encapsulation approaches exist, classified based on the encapsulation technique and the type of shell material used (Table 2). Common shell materials include amphiphilic ligands, hydrophilic inorganic substances, and water-soluble polymer matrices. This method often utilizes a wide range of both natural and synthetic biodegradable polymers, such as Polysaccharides [154], Alginate [155], Polyaspartate [156], Poly(lactic acid)-co-PEG (PLA-co-PEG) [157], Chitosan [158], polystyrene-co-PEG (PS-co-PEG) [159], PEG [155], co-polymers such as poly(maleic anhydridealt-1-octadecene)−PEG [160], and inorganic material such as silica [161] can be used. For example, PEG is one of the polymers which has phospholipid, where phospholipid bound to the nanocrystal and PEG chain will be coordinated around them and make them stable in aqueous media and, after attaching to DNA, they behave like fluoresce [162,163].

**Table 2:** Frequent polymers employed for functionalization of IONPs in medical applications.

Polymers	Advantages	Application	Ref.
Polyethylene Glycol (PEG)	Biocompatible, reduces immunogenicity, enhances circulation time	Drug delivery, MRI contrast agents, hyperthermia treatment	[164]
Dextran	Biocompatible, enhances stability, reduces opsonization, faster degradation in body fluid	Drug delivery, MRI contrast agents, cell labeling	[49]
Chitosan	Biodegradable, biocompatible, enhances cellular uptake	Gene delivery, drug delivery	[165]
Polyvinylpyrrolidone (PVP)	Biocompatible, enhances stability, reduces aggregation	Drug delivery, MRI contrast agents	[49]
Polyethyleneimine (PEI)	Enhances cellular uptake, facilitates gene transfection	Gene and drug delivery, cell labeling	[165]
Poly(vinyl alcohol) (PVA)	Reduces affrication	Cytotoxicity, drug delivery	[166,167]
Gelatin	Biocompatible, biodegradable	MRI contrast agents, biosensing	[168]
Alginate	Biocompatible, biodegradable, enhances stability	Drug delivery, wound healing, tissue engineering	[164,169]
Hyaluronic Acid	Biocompatible, enhances cellular uptake, reduces immunogenicity	Drug delivery, tissue engineering, wound healing	[49]

### 3.3. Silanization

Silica is one of the most frequently used compounds for coating the surface of IONPs to reduce their toxicity [169]. Its application is common in functionalizing nanoparticle surfaces, improving stability in water, and providing protection under acidic conditions [2]. Coating with silica typically increases the particle size and modifies the magnetic properties of IONPs. It also enables the attachment of various surface ligands and acts as a protective shield for both drug molecules and the nanoparticle itself [170]. Additionally, small compounds like pharmaceuticals, dyes, or quantum dots can be embedded into the silica layer during its formation. The silica surface allows covalent bonding with ligands and biomolecules, facilitating targeted delivery to specific organs via antibody–antigen interactions [171]. Moreover, silica coating enhances colloidal stability and allows for relatively simple control over the process, while also potentially minimizing the toxicity of IONPs.

An alternative silanization method involves coating IONPs with aminosilane (AmS), which is widely employed as a flexible drug delivery system. Surface modification of IONPs using AmS not only prevents particle aggregation but also allows the introduction of specific functionalities [144]. Studies on their biocompatibility suggest that AmS-coated IONPs only affect cellular metabolic activity at higher concentrations (around 200 μg/mL), while maintaining membrane integrity [172]. However, concentrations above 200 μg/mL have been found to reduce neuron viability by 50%, regardless of the presence of a magnetic field.

## 4. Biomedical Application

For use of IONPs in biomedical applications their toxicity and biocompatibility are so important which respect to their nature, size and composition. The other parameters like biodegradability and retention time have an essential role in biomedical applications. Herein, biomedical application of IONPs is described.

### 4.1. MRI

Magnetic resonance imaging (MRI) constitutes a robust modality that is non-invasive and possesses the capability to generate images of the human anatomy with exceptional resolution, operating fundamentally on the principles of Nuclear Magnetic Resonance (NMR); however, in certain instances, the efficacy and sensitivity of the technique may be enhanced through the utilization of contrast agents. In this context, IONPs may be employed. Upon interaction with specific cellular organelles, such as endosomes and lysosomes, the contrast characteristics of these nanoparticles are modified [173,174]. The retention duration of IONPs plays a significant role in their practical applications. The operational mechanism of MRI contrast agents is predicated upon the reduction in the relaxation time of protons, which is categorized into T_1_ relaxation and T_2_ relaxation. T_1_ relaxation is defined as the duration necessary for longitudinal magnetization to attain 63% of its equilibrium value, and transverse magnetization to revert to its initial state, with a reduction in this duration resulting in an enhancement of positive contrast. T_2_ relaxation pertains to the time required for transverse magnetization to diminish to 37% of its original magnitude and longitudinal magnetization to return to zero, where a decrease in this duration fosters negative contrast. It is imperative to note that the external magnetic fields employed in MRI typically operate at strengths of 1.5 and 3 Tesla. In this context, the dimensions, morphology, and chemical composition of IONPs are critical factors in modulating the T values. To date, IONPs have been predominantly utilized as T2 contrast agents [175,176]. Lee et al. conducted a comprehensive investigation into the varying compositions of ferrite in relation to the T value. The metal ferrites MFe_2_O_4_ (where M = Fe, Ni, Co, Mn) exhibited enhanced efficacy in the detection of cancer biomarkers when conjugated with antibodies, thereby significantly augmenting the sensitivity of MRI [177]. Smolensky et al. conducted a comprehensive investigation regarding the influence of IONP size and morphology on their relaxation properties and magnetic characteristics. The researchers performed a comparative analysis of magnetite nanocrystals exhibiting both faceted and spherical geometries, which were synthesized through thermal breakdown methods. Their findings indicated that an increase in the size of faceted nanoparticles correlates with a notable enhancement in relaxivity. Conversely, for spherical nanoparticles, no significant relationship between size and relaxivity was observed. In summary, faceted nanoparticles demonstrate superior relaxivity in comparison to their spherical counterparts [178]. Given that approximately 60% of the adult human body is composed of water, the formulation of IONPs assumes critical significance. For instance, Hu et al. documented the identification of colon carcinoma xenografts implanted in immunocompromised murine models utilizing IONPs that were functionalized with PEG. This coating facilitated the evasion of the reticuloendothelial system (RES) by the IONPs [179]. Utilizing a comparable methodology, Hurley et al. integrated IONPs with a mesoporous silica shell, which effectively inhibits the aggregation of nanoparticles while providing colloidal stability, ultimately resulting in enhanced contrast of the nanoparticles [180]. In a separate investigation, Bigall et al. synthesized magnetic nanobeads composed of hollow iron oxide through a coprecipitation process in conjunction with an amphiphilic polymer, which demonstrated a marked increase in effective magnetic anisotropy when compared to individual nanoparticles. Consequently, these nanobeads represent a superior candidate for selective negative contrast enhancement agents due to their exhibiting elevated MRI relaxivity [181,182].

Recent advancements in IONPs for MRI applications have demonstrated encouraging outcomes. Researchers are investigating exceedingly diminutive IONs measuring less than 5 nm, which are emerging as viable T_1_ contrast agents, thereby addressing the constraints associated with conventional T_2_ agents [183]. Furthermore, these nanoparticles can be utilized in the imaging of inflammatory conditions and infections [184], such as visualizing integrin αvβ3 as a specific biomarker indicative of IgA nephropathy [185], or in the detection of infections through the tracking of macrophages [186], and the diagnosis of liver fibrosis via collagen-targeting IONPs possessing a single-nanometer core size [187]. Additionally, surface ligands endowed with responsive functionalities including light, pH, enzymes, and glutathione have the capacity to modify the aggregation behavior of IONPs, as well as their hydrophilic and magnetic characteristics under varying environmental conditions [188,189,190]. Consequently, a rational design approach may be employed not only to augment the tumor accumulation of IONPs but also to finely tune the imaging signals (encompassing the imaging modality) of the tumor, resulting in a significant enhancement of tumor image contrast [191,192]. Moreover, IONP-based platforms exhibit potential in surmounting the blood–brain barrier (BBB) for central nervous system (CNS) imaging applications [96]. The functionalization of IONPs with dextran for the imaging of cerebral, peripheral, and coronary microvessels demonstrated superior outcomes compared to gadolinium-based contrast agents [193]. The biocompatible design of the functionalization of IONPs, alongside their imaging efficacy, may provide significant benefits across a diverse array of preclinical and clinical applications in MRI.

### 4.2. Targeting Drug Delivery

**Figure 9:**
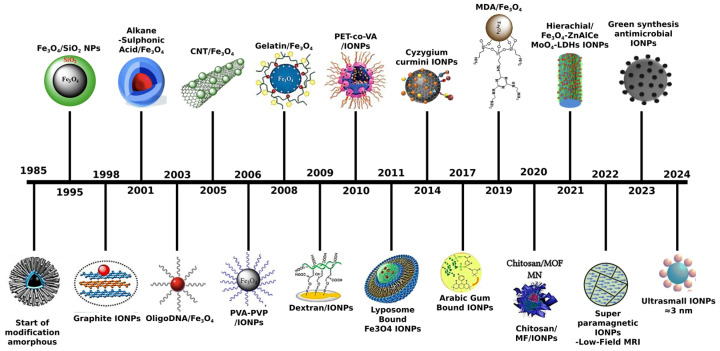
The timeline of magnetic nanoparticles in therapeutic and imaging applications. Reprinted from reference [212].

IONPs are increasingly recognized in the domain of oncology due to their unique magnetic properties and their compatibility with biological systems. These nanoparticles can be specifically engineered for various functions, with targeted drug delivery being a principal application that significantly enhances the efficacy of cancer treatments. Through their interactions with biomolecules at the cellular level, IONPs facilitate the precise targeting of malignant cells, thereby minimizing damage to surrounding healthy tissues. Furthermore, IONPs enable the magneto-mechanical stimulation of cell surface receptors, which amplifies therapeutic outcomes through methodologies such as magnetic hyperthermia, wherein localized thermal effects lead to the apoptosis of tumor cells. Typically, when pharmaceuticals are disseminated throughout the body via oral administration or intravenous injection, only a minimal fraction reaches the diseased organ. By employing targeted drug delivery, the concentration of the therapeutic agent within the specific tissues is augmented, and the crossing of physiological barriers within the body is facilitated through various active targeting strategies [194]. In particular, within the domain of oncological therapies, IONPs employed in pharmacological delivery systems represent a dependable modality. Consequently, traditional chemotherapeutic approaches may exhibit a reduced incidence of adverse effects, as IONPs facilitate the targeted administration of therapeutics and diminish the requisite dosages thereof [195]. Modified IONPs can function as carriers, facilitating the delivery of various pharmacological agents throughout the organism (Figure 9). To achieve this objective, IONPs will be encapsulated within a biocompatible, functionalized shell. In this context, the dimensions of IONPs are critically significant; they must be sufficiently diminutive to traverse vascular structures while exceeding 10 nm in size to prevent rapid systemic clearance. Consequently, the optimal size range should be established between 10 and 100 nanometers [196]. The release of the carrier at the designated target site can be triggered by internal stimuli such as alterations in pH, enzymatic activity, or external stimuli like magnetic fields, ultrasound, or light [197,198]. In order to prevent the unintended distribution of pharmacological agents to adjacent healthy tissues and to minimize the required drug dosage by enhancing its local concentration at the target site, various strategies have been proposed, including the application of an external magnetic field to precisely deliver the drug [199]. The utilization of IONPs extends beyond mere drug delivery; they are also applicable in the field of gene delivery [200,201]. Consequently, Xu et al. conceptualized a core–shell architecture that encompassed an IONPs core and a multifunctional layer, which consisted of a combination of PEG, PEI, and polysorbate 80 that encapsulated doxorubicin (DOX) as the shell. They conducted a comparative analysis of DOX uptake in cells, observing a significantly enhanced uptake of the DOX-loaded multifunctional nanoparticles in comparison to the free drug. Furthermore, they demonstrated effective magnetic targeting by employing an external magnetic field, successfully directing the drug to the tumor site and inducing apoptosis via the activation of the caspase-3 pathway [202]. To expedite drug delivery to the tumor site, Park et al. explored an innovative approach. They synthesized a nanocomposite of polymer-IONPs by covalently attaching Fe_3_O_4_ to the surface through the polymerization of heparin. This novel structure exhibited excellent dispersion in serum medium, and its magnetic field gradient effect facilitated rapid translocation across barriers, allowing for high-concentration internalization within tumor cells [203]. In the realm of gene delivery, the BBB poses significant limitations to treatment and diagnostic procedures [204]. Therefore, Kim et al. developed a novel gene delivery vehicle characterized by high efficiency. They designed a magnetofection vector composed of PCL/PEI-IONPs and oleic acid, which served to stabilize the IONPs. The encapsulation of PEI with IONPs enhanced the dispersion of IONPs and promoted the condensation of nucleic acids subsequent to their internalization within the cells [182]. Moreover, IONPs enhance the potential for controlled drug delivery through customizable surface functionalization, offering high drug-loading capacities and the possibility for targeted, magnetic-guided delivery. In recent studies, including the encapsulation of doxorubicin, IONPs demonstrate superior stability and controlled release profiles. This multifunctionality of IONPs makes them novel candidates in drug delivery systems, offering more effective and safer therapeutic options, especially in oncology, where precision and minimized side effects are critical [79]. Palake et al. introduced manganese-doped iron oxide nanoparticles (MnIONPs) as a promising dual-function platform for cancer therapy, uniquely combining magnetic targeting with chemotherapeutic delivery. In recent studies, these MnIONPs have demonstrated enhanced cellular uptake and drug release control within 3D breast cancer models, enabling the precision targeting of tumors. This innovative approach highlights the potential of MnIONPs to improve treatment specificity and therapeutic efficacy, underscoring their value as advanced carriers in cancer therapeutics [205]. Additionally, another study presents a novel approach to treating hepatocellular carcinoma (HCC) through the design of arsenic trioxide (ATO)-loaded IONPs camouflaged with HCC cell membranes, termed AFN@CM. This innovative drug delivery system enhances the efficacy of ATO by promoting ferroptosis in HCC cells, as indicated by the inhibition of glutathione peroxidase 4 and the accumulation of lipid peroxides. The cell membrane coating improves tumor targeting and minimizes toxicity, offering a promising strategy for effective and safe HCC therapy [206]. Recent advancements in the use of iron oxide nanoparticles (IONPs) in imaging and therapeutic applications have shown promising results, particularly in magnetic resonance imaging (MRI) and targeted drug delivery. In 2022, researchers demonstrated that superparamagnetic iron oxide nanoparticles (SPIONs) could serve as effective T1 contrast agents for low-field MRI, enhancing image quality while offering a safer alternative to traditional gadolinium-based agents [207]. In another study, Segers and his colleagues investigated the effects of these nanoparticles in hyperlipidemic atherosclerosis models, revealing that ferumoxide and ferumoxtran increase apoptosis and reactive oxygen species in lipid-laden macrophages, while ferumoxytol does not induce such harmful effects. Importantly, these nanoparticles were linked to enhanced inflammation and cell death in both murine models and human carotid artery plaques, suggesting that they may adversely affect disease progression in patients with advanced atherosclerosis [208]. In 2023–2024, a breakthrough study used ultrasmall iron oxide nanoparticles (USIO NPs) combined with MRI-guided focused ultrasound (MRgFUS) to improve glioblastoma imaging by crossing the blood–brain barrier, significantly boosting tumor visibility [209]. Wang and his co-workers developed a novel magnetofection system for gene delivery, demonstrating significant therapeutic potential in regulating differentiation for lumbar degenerative disk disease (DDD), a major orthopedic challenge. While intervertebral fusion is the standard treatment, its effectiveness is often lacking. Their study showed that co-stimulation with an electromagnetic field (EMF) and iron oxide nanoparticles (IONPs) significantly enhanced the delivery of therapeutic miR-21 into bone marrow mesenchymal stem cells (BMSCs) and human umbilical endothelial cells (HUVECs), promoting osteogenesis and angiogenesis. The gene-edited cells were successfully integrated into polycaprolactone (PCL) and hydroxyapatite (HA) scaffolds for tissue-engineered bone, with enhanced transfection efficiency linked to the activation of the p38 MAPK pathway, indicating this system’s promise for treating various orthopedic conditions [210]. Shanmugam and his co-workers studied iron nanoparticles, known for their diverse applications due to unique properties. They synthesized iron oxide nanoparticles (IONPs) using black cumin seed extract as a reducing and capping agent in three concentrations (1:1, 2:4, and 1:4). Characterization through UV-visible spectroscopy, XRD, FTIR, and AFM confirmed the successful formation of IONPs, with UV-visible spectra showing peak absorbances at 380 nm for 1:1, 400 nm for 2:4, and 680 nm for 1:4. AFM revealed spherical nanoparticles, while XRD indicated a cubic crystal structure. FTIR analysis showed characteristic peaks at 457.13 and 455.20 cm^−1^. Notably, the black cumin extract-mediated IONPs demonstrated significant antibacterial, antifungal, antioxidant, and anti-inflammatory activities in a dose-dependent manner [211]. These developments underscore the versatility of IONPs in advancing diagnostic precision and therapeutic delivery in oncology.

### 4.3. Tissue Engineering

The objective of tissue engineering is to fabricate functional replacement tissues that exhibit significant properties when integrated with biomaterials. In this context, the utilization of cells and the regulation of their processes pose considerable challenges within the field of tissue engineering [213,214,215]. To address this issue, the employment of magnetic cells emerges as an effective strategy. These cells can be manipulated and controlled by an external magnetic field, thereby regulating their assemblies. Ito et al. successfully established a three-dimensional culture of cells through the application of magnetic force. They developed heterotypic multilayered cell sheets that effectively enhanced the secretion of albumin in conjunction with the cells [216]. In a separate investigation, Heidari et al. synthesized a scaffold composed of IONPs, hydroxyapatite, and chitosan for the purpose of bone tissue engineering. The synthesized IONPs were characterized by a size range of 10–40 nm, with the magnetic crystal size measuring approximately 23.5 nm. Recently, the utilization of peptide-modified magnetite liposomes as selective labels for targeting cells, facilitated by computational methods, has enabled the control of cell adhesion [217]. Wang et al. present a novel magnetofection system employing iron oxide nanoparticles (IONPs) for targeted gene delivery, specifically for promoting osteogenesis and angiogenesis in lumbar degenerative disk disease (DDD). By combining electromagnetic fields (EMF) with IONPs, the system significantly enhances the transfection efficiency of therapeutic miR-21 into bone marrow mesenchymal stem cells and human umbilical endothelial cells. This innovative approach not only improves cellular responses but also activates critical pathways, demonstrating its potential as an effective therapeutic strategy for orthopedic disorders [210].

### 4.4. Stem Cell

Stem cells represent a unique category of cellular entities capable of undergoing differentiation into various cellular lineages. Embryonic stem cells exhibit pluripotency, enabling them to differentiate into all possible cell lineages [218,219]. Numerous tissues, including hematopoietic, gastrointestinal, epidermal, neural, hepatic, and mesenchymal stem cells (MSCs), also encompass stem cell populations. The propensity for self-renewal in these stem cells is comparatively lower, yet they possess the capacity to differentiate into distinct cell types, attributable to the activation of their genetic framework [218,220,221,222,223,224]. The utilization of these cells has garnered significant interest in the therapeutic intervention of various defects and diseases. The magnetic labeling of human mesenchymal stem cells utilizing IONPs facilitates the tracking of these cells through MRI [225,226,227]. Barrow et al. employed modified superparamagnetic nanoparticles composed of polyethylene glycol, polyvinyl pyrrolidone, and polyethylene imine (PEI), synthesized via thermal decomposition, to label adipose tissue stem cells. The water-soluble PEG/PVP-SIONPs and PEG/PEI-SIONPs serve as contrast agents for cell tracking via MRI. Their investigation demonstrated that PEG/PEI-SIONPs exhibited superior labeling efficiency in comparison to commercial IONPs, and upon evaluating these two modified nanocomposites, PEG/PEI-SIONPs were found to possess enhanced labeling efficiency relative to PEG/PVP-SIONPs following cellular uptake [228]. Furthermore, Skelton et al. utilized ferumoxytol as IONPs to label human embryonic stem cell-derived cardiac progenitor cells (hESC-CPCs) with the objective of enabling rapid tracking via MRI. Their findings indicated that IONPs exerted no detrimental effects on cell viability and additionally improved the localization tracking of IONPs that were anchored to hESC-CPCs implanted within porcine cardiac tissue. Consequently, they concluded that IONPs could serve as differentiation agents [229]. In the context of myocardial infarction, the paracrine action of mesenchymal stem cells has demonstrated therapeutic efficacy. In this context, the interaction between mesenchymal stem cells and cardiac cells plays a critical role, facilitated by the formation of active gap junctions. Accordingly, Han et al. co-cultured MSCs with IONPs anchored to H9C2 cells, which are characterized by gap junction protein expression, and observed the establishment of active intercellular connections with H9C2 cells, which holds promise for the repair of myocardial infarction. In this study, IONPs played an indispensable role in fostering the development of gap junctions between cells through active intracellular signaling cascades. Moreover, IONPs exhibited potential in generating cardiomyocyte-like stem cells (cMSC) from MSCs [230]. Jung et al. elucidates an innovative magnetofection system utilizing IONPs to enable the precise delivery of therapeutic miR-21, thereby augmenting osteogenesis and angiogenesis in the management of lumbar degenerative disk disease. The synergistic application of electromagnetic field (EMF) stimulation alongside IONPs markedly enhances transfection efficacy, thereby activating pivotal signaling pathways such as p38 MAPK. This groundbreaking methodology signifies a promising avenue for gene therapy within orthopedic contexts, highlighting the considerable potential of IONPs in ameliorating therapeutic results [231]. In an alternative investigation, IONPs are utilized as an innovative therapeutic approach, capitalizing on their distinctive magnetic characteristics and biocompatibility to optimize drug delivery mechanisms. The researchers adeptly incorporate IONPs into pharmaceutical formulations, exhibiting enhanced pharmacokinetic profiles and targeted therapeutic effects, which substantially augment therapeutic efficacy while concurrently reducing adverse effects. This pioneering application underscores the potential of IONPs not merely as drug carriers but also as active components in diagnostic imaging and therapeutic practices. The capacity to manipulate their physical and chemical attributes facilitates the conception of sophisticated therapeutic systems customized to specific clinical requirements, positioning IONPs as a compelling candidate in the advancement of next-generation biomedical innovations [232].

### 4.5. Biosensors and Diagnostic Tools

In comparison to techniques that do not utilize magnetic nanoparticles (MNPs), methods that employ MNPs exhibit advantageous enhancements regarding sensitivity, limit of detection (LOD), signal-to-noise ratio, and response time. The material Fe_3_O_4_ serves as a significant biosensor substrate due to its superparamagnetic characteristics and its compatibility with biological tissues. Within biological solutions, Fe_3_O_4_ experiences attraction to magnetic dipolar forces, leading to potential aggregation into clusters attributed to its substantial surface area to volume ratio. This challenge can be mitigated through the functionalization of Fe_3_O_4_, thus enhancing its compatibility with living tissues. Magnetic nanoparticles find applications across various biosensor modalities, including electrochemical, electroluminescent, solid-state nanopore, and optical biosensors. In the subsequent sections, we will investigate a selection of these biosensor technologies.

#### 4.5.1. Electrochemical Biosensor

Electrochemical biosensors represent sophisticated analytical instruments that amalgamate biological recognition components with electrochemical transduction mechanisms to identify a diverse array of analytes [233]. These sensors provide highly sensitive, cost effective, and rapid methodologies for applications in environmental monitoring, food safety, and clinical diagnostics [234,235,236]. Recent developments encompass the incorporation of nanomaterials aimed at enhancing sensitivity and enabling multi-detection capabilities [234], alongside the innovation of portable and wearable devices [237]. The architecture of electrochemical biosensors can be predicated upon potentiometric, amperometric, or impedimetric transducer systems [234,238]. The domain has experienced notable advancements in enzyme-based amperometric biosensors, screen-printed electrodes, and paper-based sensor technologies [237,238]. Furthermore, machine learning methodologies have been utilized to analyze extensive sensing datasets and enhance sensor functionality [239]. Recent research has focused on developing electrochemical biosensors that incorporate iron oxide nanoparticles (IONPs) for diverse applications, including Chronoamperometry (CA), Electrochemical Impedance Spectroscopy (EIS), Cyclic Voltammetry (CV), Chronocoulometry (CC), Differential Pulse Voltammetry (DPV), Tuberculosis (TB) detection, Organophosphorus Pesticides (OPs) analysis, Ochratoxin A (OTA) monitoring, Circulating Tumor Cells (CTCs) detection, Surface-Enhanced Raman Scattering (SERS), and Photoelectrochemical (PEC) methods (Table 3). These biosensors have demonstrated considerable potential in the detection of organic chemicals like dopamine [240], DNA [241,242], and glucose [243].The integration of IONPs with materials such as nanocellulose and graphene facilitates the electron transfer and improves the performance of the biosensor [244]. The compatibility of IONPs with hydrogels has been systematically examined for prospective biosensor applications [245]. In addition, iron-based magnetic nanoparticles have been investigated for monitoring food safety, presenting merits such as stability and environmentally sustainable properties [246]. Collectively, electrochemical biosensors based on IONPs exhibit significant potential for diverse analytical applications owing to their sensitivity, selectivity, and economic viability. The carbon electrode underwent modification with a composite of magnetic nanoparticles as reported by Gan et al. [247]. The apparatus employed Fe_3_O_4_/Au magnetic nanoparticles coated with acetylhydrolase, which were affixed to a modified screen-printed carbon electrode that integrated carbon nanotubes, nano-ZrO_2_, Prussian blue, and Nafion. The detection of dimethoate concentrations was conducted utilizing a biosensor, with the outcomes aligning closely with those derived from a gas chromatographic flame photometric detector (GC-FPD). The findings indicated that the biosensor, which incorporated conductive Fe_3_O_4_/Au MNPs, presented considerable advantages in the detection of organophosphorus pesticides, owing to its expansive surface area which enhances the sensitivity towards thiocholine (TCh). 

The electrochemical impedance immunosensor innovated by Zamfir et al. is capable of detecting ochratoxin-A through the application of anti-ochratoxin-A monoclonal antibodies integrated into carboxylated MNPs at a gold electrode interface. MNPs that have undergone carboxyl modification exhibit favorable regeneration properties, as well as a reduction in sensor impedance, thereby enhancing the LOD [248]. Furthermore, such MNPs can be utilized for the immobilization of monoclonal antibodies, including anti-ochratoxin-A monoclonal antibodies. In a recent investigation, Martin and colleagues introduced a novel electrochemical biosensor predicated on poly(dopamine) molecular nanoparticles synthesized in situ via a core–shell Fe_3_O_4_@poly(dopamine) MNP methodology [249]. In a study by Shamsipur et al., a sandwich-type immunoassay utilizing a silver signal enhancement strategy was employed for the highly sensitive detection of HER2. This method involved the functionalization of magnetite nanoparticles coated with 3-aminopropyltrimethoxysilane (APTMS) with anti-HER2 antibodies, forming a platform bioconjugate (PB). Using this approach, HER2 concentrations were quantitatively detected within a range of 0.5 pg/mL to 50 ng/mL, with a limit of detection (LOD) reaching as low as 20 fg/mL [250]. The device conceptualized by Benvidi and Jahanbani represents an electrochemical biosensor employing modified carbon paste electrodes (MBCPEs), enhanced with silver nanoparticles (Ag NPs) and Fe_3_O_4_. The MBCPE/Fe_3_O_4_@Ag nanocomposites were employed for the detection of DNA, demonstrating exceptional conductivity, a high surface area to volume ratio, notable linearity, and a low LOD for their biosensor. Their biosensor exhibited rapid response times, long-term stability, cost-effectiveness, and high sensitivity [251].

#### 4.5.2. Optical Biosensor

Optical biosensors have emerged as formidable instruments for the detection of biological systems, characterized by their exceptional sensitivity, swift response times, and the potential for seamless integration [252]. These sensing devices employ a diverse array of technologies, encompassing surface plasmon resonance, optical waveguides, and photonic crystals [252,253]. IONPs have emerged as multifaceted instruments in the realm of biosensing applications owing to their distinctive properties. The integration of IONPs into optical biosensors presents a significant advancement in biosensing technology. Their ability to enhance the refractive index and amplify signals, particularly in SPR-based systems, demonstrates their potential in achieving higher detection sensitivities [254,255]. The combination of superparamagnetic properties and optical characteristics allows IONPs to improve the performance of various optical biosensing platforms, making them indispensable tools in the detection of biomolecules [256]. Xue and colleagues effectively synthesized polyethyleneimine-capped IONPs utilizing a solvent under elevated temperatures and pressures, subsequently facilitating the self-assembly of gold nanoparticles onto a substrate. In addition to 4-mercaptobenzoic acid, the rBSA compound underwent modification with folic acid to enhance specificity towards HeLa cells. The resultant composites exhibited an exceptional LOD for circulating tumor cells, as determined by surface-enhanced Raman scattering (SERS) technology [257]. In a separate study, a novel methodology was established for the fabrication of amino-modified fluorescent magnetic nanocomposite materials through the integration of astragalus polysaccharide, Fe_3_O_4_ nanoparticles, and CdTe quantum dots. These composites were employed in the detection of trace amounts of target DNA, characterized by high sensitivity, straightforward operational procedures, facile enrichment, and convenient separation via magnetic means [258]. Jie and associates determined a method to reduce the production costs associated with an electrochemiluminescent Fe_3_O_4_@CdSe nanocomposite, which was subsequently utilized for the detection of thrombin through a DNA cycle amplification technique. A subsequent investigation implemented a surface imprinting technique in conjunction with precipitation polymerization to synthesize composites comprising Fe_3_O_4_ nanoparticles and molecularly imprinted polymers (MIPs). Employing fluorescein as a fluorescent indicator enabled the selective and sensitive detection of 17β-estradiol. Ultimately, the detection methodology proved to be straightforward, rapid, convenient, environmentally benign, accurate, and cost-effective [259]. As research in this field continues to evolve, IONPs are expected to play an increasingly prominent role in the development of next generation biosensing technologies, driving innovations in medical diagnostics, environmental monitoring, and beyond.

#### 4.5.3. Field-Effect Transistor Biosensor

Field-effect transistor (FET) biosensors have emerged as powerful tools for the rapid, highly sensitive, and label-free detection of a broad spectrum of biomarkers [260]. These devices provide notable benefits, including ease of miniaturization, seamless integration into systems, and the ability to support high-throughput screening [261]. Recent innovations in FET biosensor technology encompass advances in interface engineering, the use of nanomaterial-based transducers, and the incorporation of novel biorecognition elements [262,263]. FET-based biosensors incorporating nanomaterials, such as IONPs, offer remarkable sensitivity, selectivity, and the potential for miniaturization [262,264]. These nanoparticles can be functionalized with enzymes or antibodies for specific target detection [265]. Moreover, integrating IONPs with zinc oxide nanorods or SnO_2_ nanowires enhances biosensor performance by increasing stability, surface roughness, and sensitivity of the FET. These devices have been successfully developed for the detection of various biomolecules, including glucose [266], cholesterol [267], and ethanol [268]. While challenges related to fabrication and signal amplification persist, ongoing advancements in IONP-based FET biosensors hold significant potential for future point-of-care diagnostics and biomedical applications [269].

#### 4.5.4. Nanopore Biosensor

Nanopore biosensing is a rapidly advancing field with diverse applications in molecular detection and analysis. These single-molecule sensors offer label-free, high-throughput detection of various biomolecules, including DNA, RNA, and proteins [270,271], and can be biological, solid-state, or hybrid, each with unique advantages [272]. Nanopore sensing has shown promise in disease diagnosis, detecting biomarkers, and cancer [273]. Beyond DNA sequencing, nanopores are being explored for single-protein analysis, chemical catalysis, and biophysical characterization [274,275]. Recent advances in nanopore biosensing have incorporated IONPs to enhance detection capabilities. In electrolyte solutions, the Resistive Pulse Sensing (RPS) technique employs the Coulter principle for the enumeration and sizing of particles. RPS demonstrates enhanced sensitivity and throughput, rendering them appropriate for applications in domains such as biological research and medical diagnostics, among others [276]. Recent investigations have primarily focused on the detection of DNA through Resistive Pulse Sensing by observing the adsorption of DNA to particles. The binding of thrombin molecules to DNA-functionalized beads results in alterations to the overall size and surface charge of the NPs/DNA/thrombin complex. Consequently, thrombin detection relies on the amplitude and Full Width Half Maximum (FWHM) [277], as well as variations in the FWHM induced by the annealing or hybridization of target DNA to probes that possess complementary DNA sequences affixed to magnetic particles. Hao Wang successfully integrated Fe_3_O_4_/Au core–shell nanoparticles, functionalized with peptide nucleic acid, into quartz capillaries with a 20.0 nm diameter tip to specifically target miRNA in complex samples for the precise detection of single microRNA molecules [278]. Kyloon Chuah and colleagues engineered cost-effective silicon nitride solid-state nanopore systems for protein detection within complex samples.

**Table 3:** IONPs employed in electrochemical biosensors.

Type of Sensor	Target	Limit of Detection	Dynamic Range	Ref.
Electrochemical biosensor				
CA ^a^	Carcinoembronic antigen	-	4–25 ng/mL	[279]
CV ^b^	Glucose	0.5 mM	0.5–22 mM	[280]
CA	Proteins	750 nM	25 μM–2.0 mM	[281]
CC ^c^	miRNA	100 fM	100 fM–1.0 μM	[282]
DPV ^d^	Human papillomavirus (HPV)	0.1 nM	10^−4^–1 μM	[283]
DPV	Amyloid-beta oligomers (AβO)	3.4 fM	10 fM–10 μM	[284]
EIS ^e^	Paracetamol	0.3 μM	1–5 mM	[285]
CA	Glucose	0.38 µM	1–400 µM	[286]
EIS	D aminoacid (DAA)	02–0.80 μM	0.02 μM	[287]
DPV	SARS-CoV-2	0.932 pg/mL	1 pg/mL–1 µg/mL	[288]
Amperometric	Dopamine	0.001 μM	0.006–635 μM	[240]
DPV	DNA	7.96 × 10^−13^ M	1.0 × 10^−6^–1.0 × 10^−12^ M	[241]
DPV	DNA	2 aM	10 aM–1 nM	[242]
CV	Glucose	8 μM	5 × 10^−3^–30 mM	[243]
DPV	Ops	5.6 × 10^−4^ ng/mL	1.0 × 10^−3^–10 ng/mL	[247]
EIS/SPR	OTA ^i^	0.01 ng/mL	0.01–5 ng/mL	[248]
SPR ^f^	OTA	0.94 ng/mL	1–50 ng/mL	[248]
CV	Dopamine	182 nM	6.0 × 10^−7^–8.0 × 10^−4^ M	[249]
EIS	DNA	3.0 × 10^−17^	1.0 × 10^−16^–1.0 × 10^−8^	[251]
Optical biosensor				
SERS ^g^	CTCs ^j^	1 cell/mL	1–250 cell/mL	[257]
PEC ^h^	PSA ^k^	5.0 pg/mL	1.0 × 10^−11^–5.0 × 10^−8^ g/mL	[258]
Fluorescent	17β-estradiol	0.03 μM	0.10–70 μM	[259]
FET biosensor				
I–V	Glucose	12 μM	0.05–18 mM	[266]
I–V	Cholesterol	0.06 mM	0.1–60.0 mM	[267]

^a^ Chronoamperometry, ^b^ Cyclic Voltammetry, ^c^ Chronocoulometric, ^d^ Differential Pulse Voltammetry, ^e^ Electrochemical Impedance Spectroscopy, ^f^ Surface Plasmon Resonance, ^g^ Surface-Enhanced Raman Scattering, ^h^ Photoelectrochemical, ^i^ Ochratoxin A, ^j^ Circulating Tumor Cells, ^k^ Prostate-Specific Antigen.

The analyte, a prostate-specific antigen (PSA), was captured by antigen-modified magnetic nanoparticles that did not necessitate an external magnetic field. A magnetic field was utilized to manipulate these entities, obstructing a nanopore array to prevent their translocation. A limit of detection of 0.8 fM was successfully attained [289]. Nanopore sensing integrated with nanoparticle conjugates has shown promise in single-molecule binding assays [290], and sensitive detection of single-nucleotide polymorphisms [291], offering potential for early disease diagnosis and prevention.

## 5. Challenges and Futures Perspective

The domain of IONPs is experiencing a significant evolution, characterized by notable progress in their synthesis, functionalization, and biomedical applications. As research advances, various viewpoints regarding their future prospects and associated challenges are emerging, including advancements in functionalization and targeting recent developments in IONP chemistry, which have enabled meticulous control over their dimensions, shapes, compositions, magnetization, and surface charges. These tailored properties are vital for enhancing their biocompatibility and therapeutic effectiveness. Such precision allows for the creation of theranostic agents capable of targeting, imaging, and treating diseases, especially cancer. This dual functionality facilitates real-time monitoring of therapeutic outcomes, paving the way for personalized medicine. Despite encouraging results from animal studies, translating IONPs into effective treatments for humans remains a significant hurdle. Critical factors such as particle size, shape, substitution levels, dosage, and long-term biocompatibility need thorough investigation. Addressing toxicity concerns, particularly related to cancer therapy and multi-drug resistance, is crucial. Comprehensive in vitro and in vivo testing, along with stability evaluations, are necessary to ensure the safety and effectiveness of IONPs before they are broadly implemented in clinical practice. Regulatory and multidisciplinary approaches are crucial. With the expansion of nanotechnology, establishing regulatory guidelines is essential for the safe application of IONPs in medicine. Collaboration between regulatory bodies and researchers is required to develop standardized procedures for clinical and preclinical trials. These guidelines will promote innovation while ensuring public safety. While the versatility of IONPs in drug delivery is well-established, understanding their mechanistic actions remains a critical area for future research. Exploring how nanoparticle size and synthesis conditions affect efficacy can lead to the development of more effective and safer therapeutic systems. Research into green hybrid systems that utilize tunable IONPs with optimized physicochemical and magnetic properties holds considerable promise.

## Figures and Tables

**Table 1:** Overview of functionalization techniques for IONPs.

Functionalization Strategies	Type of Material	Advantages	Disadvantages	Ref.
Ligand exchange	Functional molecules like thiols and amines	Improves water solubility, stability in biological environments, versatile ligand choices	Complications regarding stability over extended durations, necessitating meticulous regulation of ligand concentration	[138,139]
Encapsulation	Organic encapsulation: polymers, citrates	Enhances biocompatibility, stability, and dispersibility in water	Influence magnetic characteristics, possibility for large particle dimensions	[140,141]
	Inorganic-encapsulation: Au, metal oxide	Provides strong stability and inert surface for further functionalization, enhances biocompatibility	More complex synthesis, potential for reduced surface reactivity	[142,143]
Assembly	Self-assembly: Biomolecules like peptides and DNA	Precise control over structure, allows for functional complexity	Require specific environmental conditions for stability	[138]
	LbL assembly: polyelectrolyte deposition consecutively	Layered structure allows precise control over thickness and function, high versatility	Time-consuming and complex, risk of layer separation	[141]
Silanization	Silica, aminosilan	Increases colloidal stability, good for functionalization, enhances biocompatibility	Thick silica coating may reduce magnetic response, complex procedure	[144,145]
Targeting agent	Antibodies, peptides, small molecules	Enables selective targeting of specific cells or tissues (e.g., tumors)	Functionalization may reduce stability or induce immune response	[141,146]
Host-Gust strategy	Cyclodextrins, Curcurbits	Facilitates reversible binding, good for drug delivery applications	Requires precise control of host-guest interactions, limited to specific ligand types	[147]
click-chemistry	Azides, alkynes	Enables highly selective and bioorthogonal reactions, fast reaction speed	Requires specific reactants, some click-chemistry reactions can be toxic or sensitive	[141]

## Data Availability

No new data were created or analyzed in this study. Data sharing is not applicable to this article.
